# Effects of hypersensitivity disorders and environmental factors on the equine intestinal microbiota

**DOI:** 10.1080/01652176.2020.1745317

**Published:** 2020-03-30

**Authors:** S. Kaiser-Thom, M. Hilty, V. Gerber

**Affiliations:** a Swiss Institute of Equine Medicine (ISME), Department of Clinical Veterinary Medicine, Vetsuisse Faculty, University of Bern, and Agroscope, Bern, Switzerland; b Institute for Infectious Diseases, University of Bern, Bern, Switzerland

**Keywords:** Horse, equine, intestinal microbiota, allergy, culicoides hypersensitivity, severe equine asthma, environment

## Abstract

**Background:**

Recent evidence suggests that an altered intestinal microbiota, specifically a reduction of bacterial diversity or a shift in microbial composition, is associated with the development of hypersensitivity disorders in humans, but this is unknown for horses.

**Objectives:**

In this study we hypothesized that horses affected by either Culicoides hypersensitivity or severe equine asthma or both show a decreased diversity of their intestinal microbiota. We also investigated environmental effects.

**Methods:**

Rectal swab samples of a total of 140 horses were collected and the owners completed a detailed questionnaire about their horse. For each allergic horse, a healthy peer from the same stable was equally sampled as an environmentally matched control. Microbiota in the swabs was determined by assessing the V4 region of the bacterial 16S rRNA gene. Structures of bacterial communities were investigated by means of alpha and beta diversity indices.

**Results:**

Group wise comparisons between healthy and allergic horses showed no significant differences regarding alpha (p = 0.9) and beta diversity (p = 0.5). However, the microbial structure was associated with environmental factors such as the type of stable (p = 0.001), access to pasture (p = 0.001) or the type of feeding (p = 0.003). There was also a strong location effect meaning that the microbiota was more similar within the same as compared between farms within this study.

**Conclusion:**

Our observations suggest that hypersensitivity disorders in adult horses are not associated with an alteration of the intestinal microbiota, but environmental and/or location factors strongly influence these bacteria.

## 1. Introduction

There is recent evidence for a role of the intestinal microbiome in the development and maintenance of hypersensitivity disorders. The microbiota is a potent immune modulator (Geuking et al. 2011; Hooper et al. 2012; Sommer and Bäckhed 2013) and the gastro-intestinal tract has been referred to as the largest immunological organ of the body (Kelly and Coutts 2000; Wershil and Furuta 2008). The microflora hypothesis as an expansion of the well-known hygiene hypothesis (Strachan 1989) suggests that a dysbiosis of the intestinal microbiota may result in the development of hypersensitivity disorders (Noverr and Huffnagle 2005; Stiemsma et al. 2015). Especially early infancy is a critical period of microbiological and immunological development (Fujimura and Lynch 2015) and an imbalance in the bacterial composition at this time may lead to an increased risk of allergic sensitization (Legatzki et al. 2014; Rutten et al. 2015). In human medicine this has been shown for several allergic disorders, like asthma, rhinitis or atopic dermatitis (Bottcher et al. 2000; Bisgaard et al. 2011; Abrahamsson et al. 2014; Marsland et al. 2015; Zheng et al. 2016). Furthermore, children with atopic dermatitis are also at an increased risk to subsequently develop other manifestations of hypersensitivities, a phenomenon, which is known as the allergic march (Linneberg 2008; Spergel 2010; Nissen et al. 2013). But there is also evidence for an association of allergic disorders and an altered intestinal microbiota in adults. In a large-scale study with a total of 1879 fecal samples provided by the publicly available American Gut Project, Hua et al. (2016) showed that adults with allergies, especially to nuts and seasonal pollen, also have a lower microbial diversity in their feces than healthy persons.

In horses, few studies using next-generation sequencing technologies for the investigation of the intestinal microbiome are available at this time and none have focused on the association of the intestinal microbiota with equine hypersensitivity disorders. The two best characterized and most common hypersensitivity disorders are Culicoides hypersensitivity (CH, also known as insect bite hypersensitivity, sweet itch or summer eczema) and severe equine asthma (SEA, formerly known as recurrent airway obstruction). CH is caused by type 1 allergic reactions against salivary gland proteins of biting midges of the genus *Culicoides* spp. and is frequently seen in certain horse breeds, such as the Icelandic Horse where the prevalence of CH can reach up to >50% in horses imported from Iceland to Europe (Schaffartzik et al. 2012). SEA is a chronic disorder of the airways, which is characterized by hypersensitivity-mediated airway inflammation and affects approximately 10-20% of adult horses living in the Northern Hemisphere (Hotchkiss et al. 2007; Ramseyer et al. 2007; Couëtil et al. 2016). Clinical signs of SEA are triggered or exacerbated by the inhalation of dust particles present in the stables, particularly those associated with hay feeding (Leclere et al. 2011). Since there is no causative treatment, the management of CH and SEA is frequently unsatisfactory and expensive. It is centered around the reduction or even complete avoidance of allergen exposure, which often needs to be complemented by medical treatment (Léguillette 2003; Cunningham and Dunkel 2008; Jackson et al. 2010; Schaffartzik et al. 2012). Interestingly, there is recent evidence for an association between the occurrence of CH and SEA. Horses suffering from SEA have an increased risk for developing CH (Kehrli et al. 2015) and, vice versa, horses with CH also have a higher risk for airway hyperreactivity (Lanz et al. 2017). We have therefore selected both diseases for our study and hypothesized that horses affected by either CH, SEA or both show a disordered intestinal microbiota in comparison to healthy stable mates.

Since environmental factors can exert strong effects on the intestinal bacterial composition, we also investigated this aspect. Such effects have been shown especially for humans (Candela et al. 2012; Song et al. 2013), for laboratory animals (Terán-Ventura et al. 2010; Campbell et al. 2012; Thoene-Reineke et al. 2014) and in several species for diet effects (Muegge et al. 2011; Albenberg and Wu 2014; David et al. 2014). Therefore, we controlled for some environmental factors, but also investigated which factors exert the most important effects on the equine intestinal bacterial diversity and composition.

## 2. Materials and methods

### 2.1. Study design

The project was designed as a case-control study in a field research environment, which means that all horses were sampled at their home stables. Three study groups were formed: horses suffering from Culicoides Hypersensitivity (group “CH”), horses suffering from severe equine asthma (group “SEA”) and horses suffering from both disorders (group “CH-SEA”). For each allergic horse of one of these three groups a healthy peer from the same stable was sampled as a control. Horses were thus matched primarily regarding housing and management. As further criteria breed, age and sex were also matched as far as possible.

All horses underwent a general examination before the sample collection that included respiratory tract and cardiac auscultation, rectal temperature, mucous membrane colour, capillary refill time and submandibular lymph node palpation. Examination and sampling were performed by the same veterinarian (SK) in all horses. Owners were asked to complete a detailed and standardized questionnaire consisting of four parts. The first part comprised general information about the horse (e.g., breed, age). The second part comprised questions about the housing, feeding and management of the horse. Part three was used to collect information about the worst clinical signs of CH that owners had ever observed in their horse. To complement the historical information, current clinical signs were recorded by the examining veterinarian (SK) and horses were then scored as previously described by Lanz et al. (2017). In brief, this score comprises five severity grades, each with an additional characteristic to the preceding one with 0) no signs of CH; 1) increased skin flaking with visible epithelial debris; 2) areas of broken hairs or alopecia from scratching; 3) indurated skin folds; 4) obvious crusts from serous exudate, but without bleeding; 5) bleeding from self-inflicted abrasions. As diagnosis of CH is based on clinical signs and a characteristic medical history (Oliveira-Filho et al. 2012), horses were classified as CH affected if their current CH-score was >0 and their history was compatible with CH. Finally, in part four the historical lung health status of the horse was assessed using the questions from the Horse Owner Assessed Respiratory Signs Index (HOARSI) questionnaire developed by Ramseyer et al. (2007). This index also refers to the worst clinical signs in the horse’s medical history and defines four categories according to the severity of respiratory signs (HOARSI 1-4: healthy, mild, moderate and severe). It has been shown that SEA affected horses can be reliably identified by their HOARSI category (Laumen et al. 2010). Accordingly, all SEA affected horses had been graded HOARSI 3 (moderate) or 4 (severe) and, in addition, all these horses had formerly been presented to the equine clinics in Bern or Zurich, where they were diagnosed with SEA based on standard examination of the lower respiratory tract.

Horses could only serve as controls if both their CH score was 0 and their HOARSI score was 1. Furthermore, horses were excluded from the study if they showed acute systemic illness on the day of sampling, if they had received antibiotics within the last 3 months, or corticosteroids within the last 4 weeks, or if a matching healthy peer was not available.

### 2.2. Sample collection

Samples were collected between June 2017 and November 2017. We focused on the summer and fall season as clinical signs of CH are exacerbated when specific biting midges (genus *Culicoides*) are most active (Sanders et al. 2011).

Rectal swabs were obtained using sterile collection swabs (Sarstedt, PS/Viscose). Swabs were inserted 12 cm into the rectum for 15 sec while rotating and gently rubbing them against the rectal mucosa. Afterwards swabs were put into their transport tubes and placed into a cold box (at 4 °C). Upon arrival at the equine clinic, swabs were immediately put at −80 °C until further processing.

### 2.3. DNA extraction, 16S rRNA amplification, purification and sequencing

DNA was extracted from the swabs using the commercial QIAamp DNA Mini Kit (Qiagen, Hilden, Germany) according to the manufacturer’s recommendations. Forward (5′-GTGCCAGCMGCCGCGGTAA-3′) and reverse (5′-GGACTACHVGGGTWTCTAAT-3′) primers (Caporaso et al. 2011) were used to amplify the V4 region of the bacterial 16S rRNA gene and an Illumina adaptor sequence was attached at the 5′ end. PCR was then run in the same way as described by Kraemer et al. (2018), including negative controls. PCR products were purified by QIAquick PCR Purification Kit (Qiagen, Hilden, Germany) and the purified products were evaluated by gel electrophoresis.

The samples were then submitted to the Next Generation Sequencing Platform at the University of Bern for indexing and paired-end 2 × 250 bp sequencing (Reagent Kit v2) on the Illumina MiSeq platform (Illumina Inc., San Diego, USA).

### 2.4. Sequence analysis

The open-source software R version 3.3.2 (R Core Team 2018) with the DADA2 package version 1.6.0 was used to process the sequences as described by Callahan et al. (2016). Specifically, forward reads were trimmed at 180 base pairs (bp) and reverse reads at 150 bp. We decided for these rather conservative borders to make sure that the Phred quality scores for all reads at any point up to the trimming borders were well over 30, which corresponds to a base call accuracy of 99.9%. Although the DADA2 algorithm is relatively robust to lower quality sequences, deliberate trimming improves the algorithm’s sensitivity to detect rare sequence variants. Reads containing ambiguous base calls or sequences with inappropriate length (<245 bp and >257 bp) were filtered out and chimeras were also identified and removed. Taxonomy was then assigned to the remaining amplicon sequence variants (ASVs) by making use of the Silva 16S rRNA reference database version 128 (Quast et al. 2013). Finally, sequences corresponding to chloroplasts, mitochondria, Archaea and Eukaryotes were removed as well (207 sequences removed = 1,7% of sequences).

### 2.5. Statistical analysis

Data were analyzed in R using the base, *vegan*, *microbiome* and *pairwiseAdonis* packages (Lahti and Shetty 2018; Martinez Arbizu 2018; Oksanen et al. 2018; R Core Team 2018).**** To ensure that sequencing depth was sufficient for all samples rarefaction curves were visually assessed at first. Based on the authors of DADA2 and others, rarefying microbiome data for statistical analyses is discouraged and was therefore waived (McMurdie and Holmes 2014; Callahan et al. 2016; Kraemer et al. 2018). For the evaluation of alpha diversity (within-sample diversity) we investigated the number of ASVs in each sample, Pielou’s evenness index and Shannon diversity. We compared the aforementioned alpha diversity indices by groups (e.g., allergic vs. healthy) using an unpaired Wilcoxon rank-sum test or a Kruskal-Wallis test as data were not normally distributed. We also performed pairwise comparisons of all three alpha diversity indices between affected horses and their matched control horses using a paired Wilcoxon test as well as a Jonckheere’s trend test for environmental comparisons since the respective subcategories can be brought into a logical order (e.g., ordered by the amount of roughage and concentrate).

Beta diversity measurements (between-sample diversity) were assessed by means of weighted (abundance-based) and unweighted (presence/absence-based) Bray-Curtis indices. Nonmetric multidimensional scaling was then performed of the generated distance matrices in order to plot dissimilarities. Differences between groups of samples were statistically evaluated by a permutational multivariate analysis of variance using the distance matrices. Post-hoc testing for multilevel pairwise comparisons was performed with the *pairwise.adonis* function. Mean dissimilarity distances within specific groups (e.g., all horses from the same stable) were investigated by compiling all appropriate pairwise distance values from the distance matrix based on weighted and unweighted Bray-Curtis dissimilarity calculations. As these data were not normally distributed, groups were compared by an unpaired Wilcoxon rank-sum test. A p-value of <0.05 was considered for statistical significance for all comparisons.

## 3. Results

### 3.1. Study population

**Figure 1.**
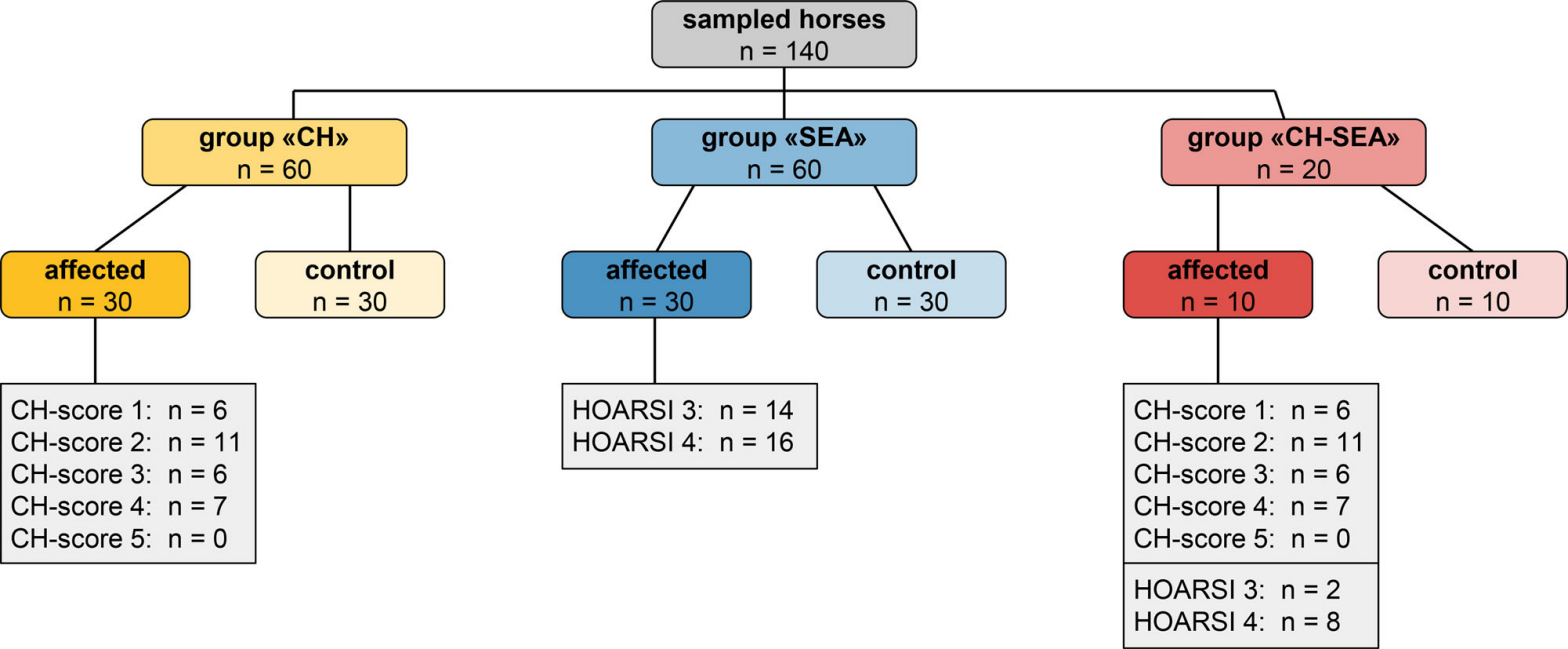
Flow chart of the study population including the distribution of CH- and HOARSI scores. CH = Culicoides hypersensitivity, SEA = severe equine asthma, HOARSI = horse owner assessed respiratory signs index.

A total of 140 horses met the inclusion criteria, 70 were affected by a hypersensitivity disorder and the other 70 were the respective healthy and stable-matched controls. Of these, three study groups (“CH”, “SEA” and “CH-SEA”) were formed (Figure 1). The horses originated from altogether 48 farms from all over Switzerland and further details about each horse can be found in the supplementary material (Additional Table A1).

**Table 1.** Comparisons of the three alpha diversity indices richness, evenness and Shannon index within several subcategories.

			*n*	Richness			Evenness			Shannon Index		
				mean and SD	*p*-value unpaired^1^	*p*-value combined^2^	mean and SD	*p*-value unpaired^1^	*p*-value combined^2^	mean and SD	*p*-value unpaired^1^	*p*-value combined^2^
**Disease**	**allergic vs. healthy**	allergic horses healthy controls	70 70	848 ± 249 820 ± 221	0.5	0.6	0.79 ± 0.06 0.80 ± 0.07	0.4	0.6	5.35 ± 0.56 5.34 ± 0.57	0.9	1.0
	**CH vs. healthy**	CH-affected horses healthy controls	30 30	887 ± 218 881 ± 252	0.8	0.8	0.81 ± 0.05 0.79 ± 0.08	0.9	0.7	5.46 ± 0.44 5.35 ± 0.68	0.7	0.6
	**SEA vs. healthy**	SEA-affected horses healthy controls	30 30	802 ± 284 775 ± 218	0.5	0.7	0.79 ± 0.07 0.80 ± 0.06	0.6	0.5	5.25 ± 0.70 5.31 ± 0.51	0.8	0.8
	**CH-SEA vs. healthy**	CH-SEA-affected horses healthy controls	10 10	873 ± 192 860 ± 237	1.0	0.9	0.79 ± 0.03 0.81 ± 0.02	0.3	0.2	5.35 ± 0.23 5.45 ± 0.32	0.5	0.6
**Environment/ Management**	**type of stable**	interior box exterior box open stable pasture only	25 24 82 9	909 ± 274 730 ± 209 875 ± 231 630 ± 155	0.003 ***	0.2 (decr.)	0.77 ± 0.10 0.79 ± 0.07 0.81 ± 0.04 0.79 ± 0.03	0.1	0.3 (decr.)	5.22 ± 0.82 5.17 ± 0.69 5.47 ± 0.39 5.08 ± 0.34	0.02 *	0.4 (decr.)
	**access to pasture**	0-4 hours per day 4-12 hours per day >12 hours per day	28 50 62	863 ± 272 880 ± 242 799 ± 228	0.2	0.05 * (decr.)	0.80 ± 0.07 0.79 ± 0.08 0.81 ± 0.04	0.8	0.3 (incr.)	5.35 ± 0.62 5.33 ± 0.65 5.36 ± 0.44	0.7	0.2 (decr.)
	**type of feeding**	roughage only roughage dominating roughage and concentrate	17 62 61	867 ± 263 872 ± 193 802 ± 280	0.1	0.04 * (decr.)	0.80 ± 0.02 0.82 ± 0.04 0.78 ± 0.08	0.001 ***	0.03 * (decr.)	5.37 ± 0.32 5.53 ± 0.38 5.16 ± 0.69	0.003 ***	0.02 * (decr.)
**Signalment**	**type of breed**	horse breed pony breed	64 76	836 ± 273 844 ± 218	0.5	–	0.79 ± 0.07 0.81 ± 0.05	0.2	–	5.27 ± 0.64 5.42 ± 0.48	0.3	–
	**gender**	male female	89 51	850 ± 261 823 ± 213	0.4	–	0.80 ± 0.07 0.80 ± 0.06	0.6	–	5.34 ± 0.61 5.37 ± 0.47	0.5	–

The unpaired comparisons (^1^) were done by means of unpaired Wilcoxon rank-sum tests or Kruskal-Wallis tests. The combined comparisons (^2^) were done by means of paired Wilcoxon rank-sum tests (within the category “disease”) or Jonckheere’s trend tests (within the category “environment/management”). CH = Culicoides hypersensitivity, SEA = severe equine asthma. Levels of significance: * *p* ≤ 0.05 | ** *p* ≤ 0.01 | *** *p* ≤ 0.001.

### 3.2. Sequences

One sample of every participating horse (n = 140) was included in the study. A total of 14’909’138 reads were recorded and these reads were clustered into 11’658 amplicon sequence variants (ASVs). The mean number of reads per sample was 106’494 (with a standard deviation of 53’494), ranging from 28’295 to 508’739 reads. A rarefaction curve was used to investigate the sequencing depth and due to the low slope of the curve, the sequencing depth was found to be sufficient (Supplementary material, Additional Figure A1).

### 3.3. Alpha diversity comparisons

We defined three root categories (“disease”, “environment/management” and “signalment”) and compared the three indices richness, evenness and Shannon index within several subcategories (Table 1). Within the category “disease” we could not detect significant differences, neither for unpaired comparisons between groups, nor for paired comparisons between the affected horses and their respective controls. The same applies for the type of breed and the sex (category “signalment”). In contrast, environment/management factors had significant influences on the alpha diversity values. Specifically, richness, evenness and Shannon indices were decreasing from irregular to daily (pasture access) and from a roughage-based diet to larger amounts of concentrate (feeding type).

We also investigated age as a factor. The horses included in this study ranged in age from 2 to 30 years (mean age: 15.6 years, ±6.5 years). Scatterplots with regression lines and 95% confidence intervals were created, but the regression lines remained almost parallel to the x axis (Supplementary material, Additional Figure A2), indicating that there was no significant change within our range of ages.

**Figure 2.**
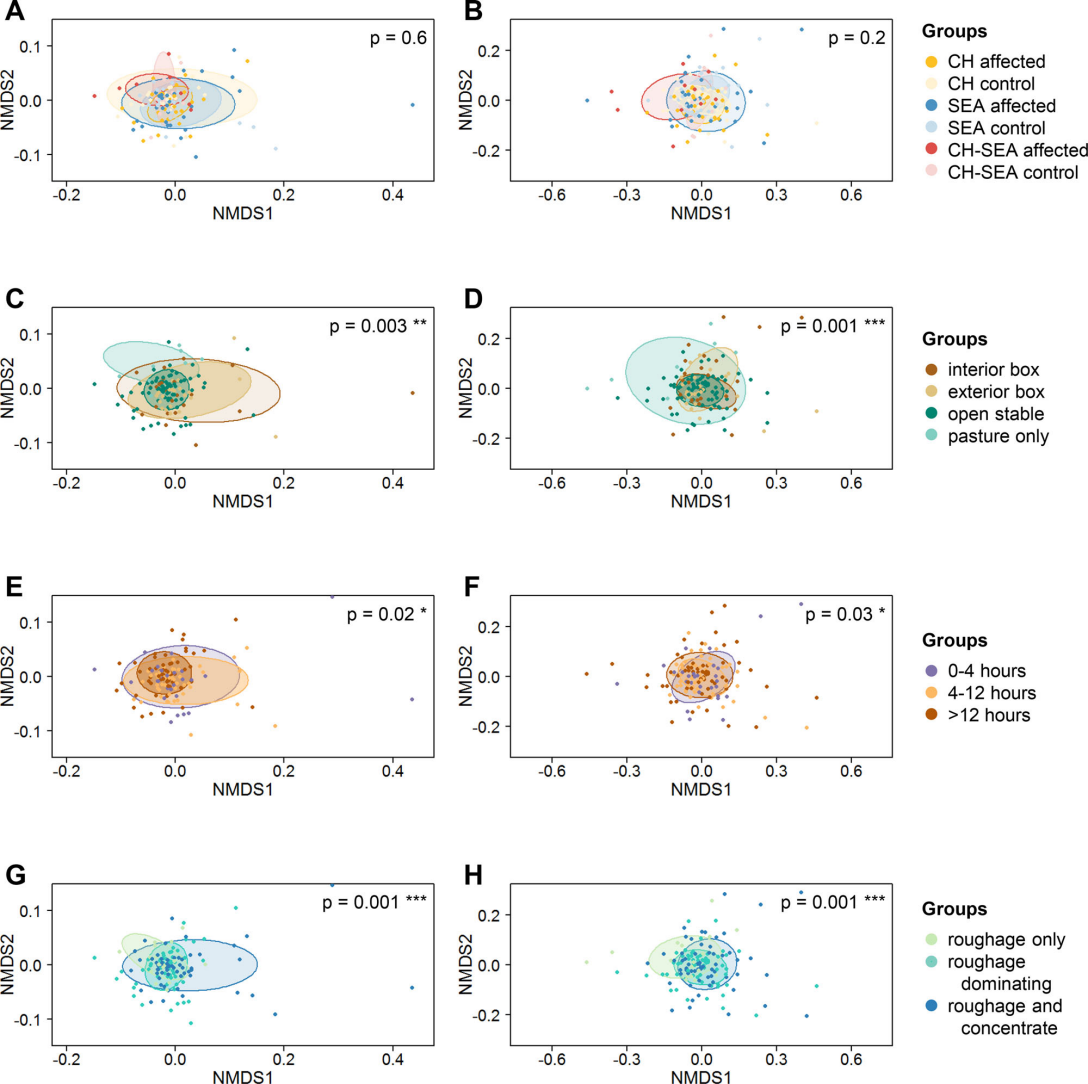
Nonmetric multidimensional scaling (NMDS) plots. The plots are based on the weighted (left side) and unweighted (right side) Bray-Curtis dissimilarity distance matrices. A and B: Comparisons of study groups (CH = Culicoides hypersensitivity, SEA = severe equine asthma). C and D: Comparisons of stable types. E and F: Comparisons of pasture access rates. G and H: Comparisons of feeding types.

### 3.4. Beta diversity comparisons

Differences in weighted and unweighted Bray-Curtis dissimilarities were tested among the same categories as described above. Again, significant differences could only be found for comparisons of the category “environment/management”. Nonmetric multidimensional scaling (NMDS) plots were created for these comparisons (Figure 2(C–D)) as well as for the comparisons of study groups (Figure 2(A–B)).

**Figure 3.**
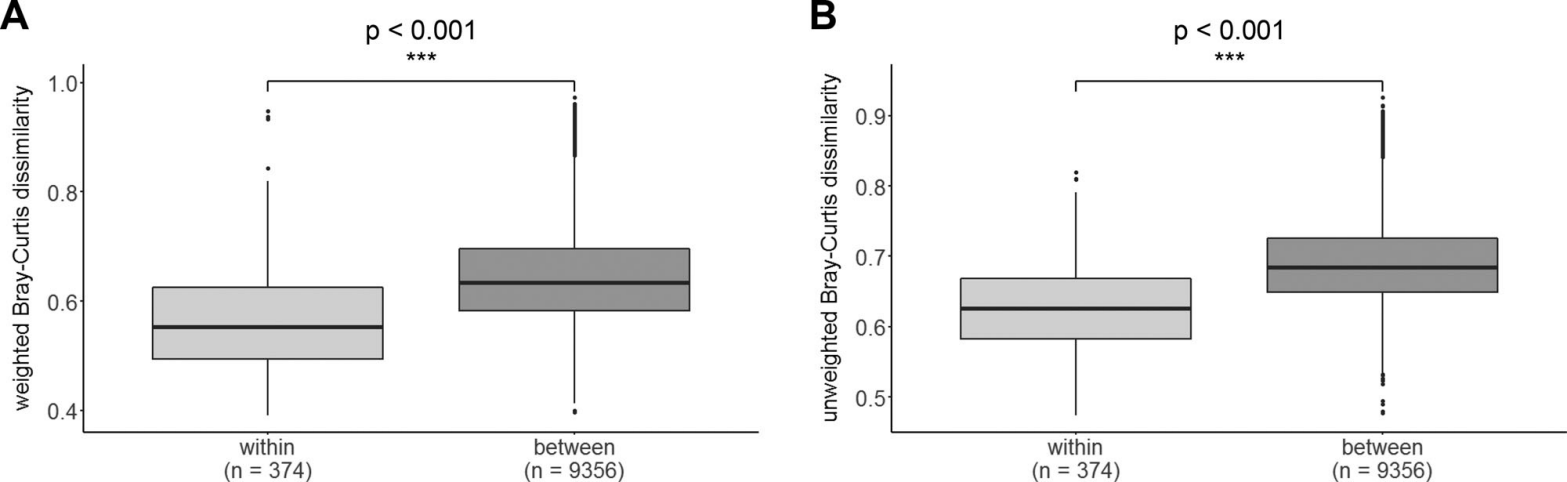
Boxplots for the investigation of a “location effect”. Calculations were made based on weighted (A) and unweighted (B) pairwise dissimilarity distances. Pairwise distances between horses originating from the same stable (“within stable”) are significantly lower than pairwise distances between horses originating from different stables (“between stables”).

In order to determine the impact of the location (stable ID) on the equine intestinal microbiota, we then compared the weighted and unweighted Bray-Curtis dissimilarity distances of pairwise combinations of horses from the same stable (“within stable”) to all pairwise distances of horses that originated from different stables (“between stables”). There was a highly significant difference (Wilcoxon rank-sum test, p < 0.001) between these two groups with “within” distances being smaller than “between” distances (Figure 3(A–B)).

### 3.5. Diversity of bacterial taxa

The majority of ASVs belonged to the phylum Verrucomicrobia (36.3% ± 9.7%), followed by Bacteroidetes (28.0% ± 10.0%), Firmicutes (21.8% ± 5.1%), Cyanobacteria (5.1% ± 3.4%) and Proteobacteria (2.8% ± 3.2%). The remaining 6% of the bacterial population belonged to one of 24 phyla, all of which made up less than 1.5% of the relative abundance each. The most frequent ASV within each of those top 5 phyla was determined (Additional Table A2), as well as the top 20 most abundant ASVs overall (Additional Table A3). Twelve of these 20 belonged to unassigned genera from Subdivision 5 of the phylum Verrucomicrobia. Even by manually checking with the online RDP sequence match tool they could not be assigned further on a species level (Cole et al. 2005).

**Figure 4.**
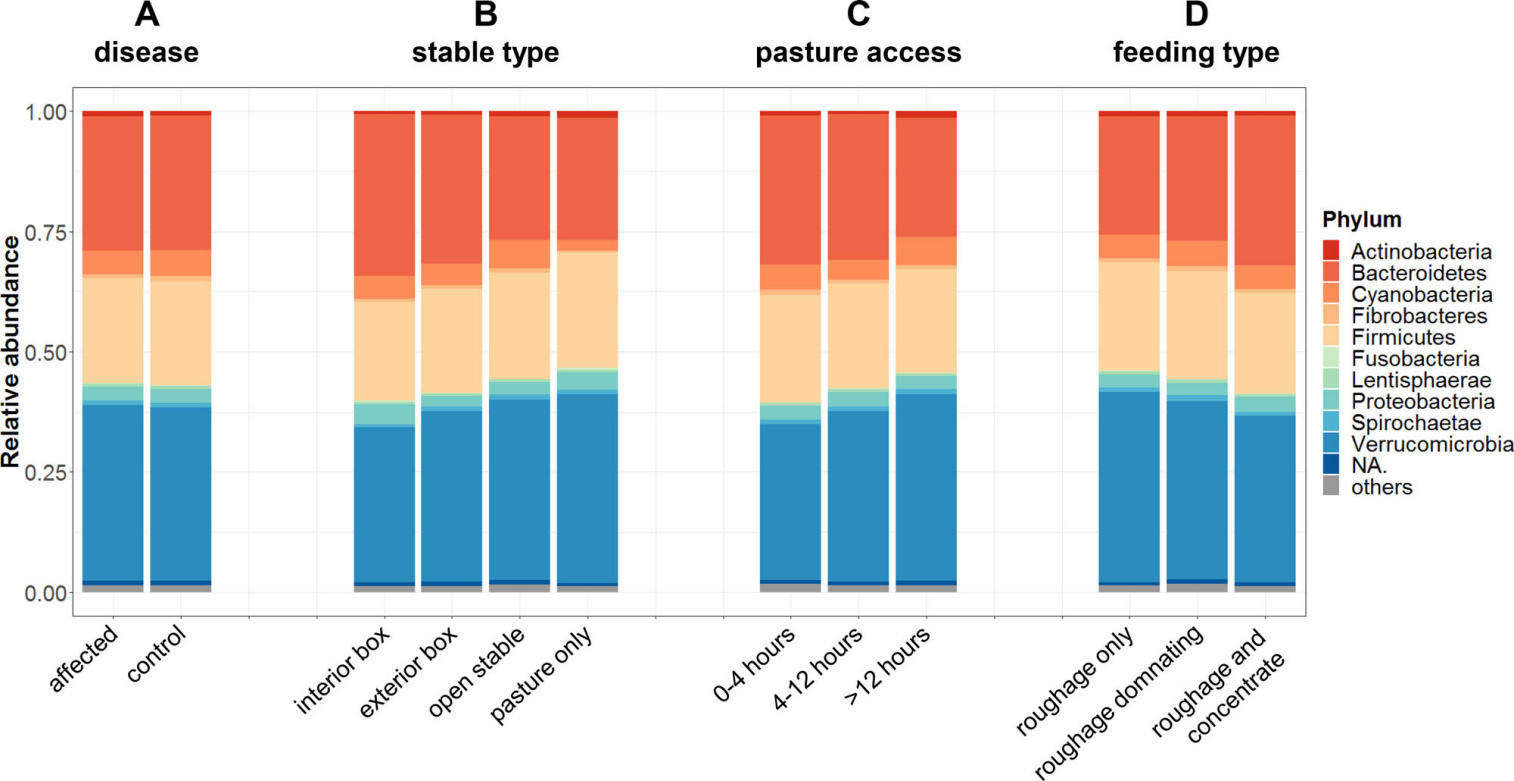
Mean percentage distribution of bacterial phyla. Relative bacterial abundances as a function of hypersensitivity disease (A), stable type (B), pasture access per day (C) and feeding type (D).

Barplots were created in order to visualize the percentage distributions of bacterial phyla for the comparison of affected vs. healthy (Figure 4(A)) and for the three environmental categories “type of stable” (Figure 4(B)), “pasture access” (Figure 4(C)) and “type of feeding” (Figure 4(D)).

## 4. Discussion

This study offers new data regarding the equine intestinal microbiota and important influencing factors. Derived from findings in human medicine, we hypothesized that the intestinal microbiota of adult horses suffering from either CH or SEA or both differs from that of healthy horses, specifically in terms of microbial diversity and composition. However, when comparing these in affected and healthy horses, we found that neither alpha nor beta diversity indices were significantly different. This may be due to the chosen age groups, since all horses were adults. Although Hua et al. (2016) could demonstrate alterations in the fecal microbiota of adult humans suffering from allergies, many other studies focus on the neonatal time period (Kalliomäki et al. 2001; Penders et al. 2007; Craig 2016), suggesting that perturbations are more likely associated with hypersensitivities when occurring in early life (Arrieta et al. 2014; Fujimura and Lynch 2015; Tamburini et al. 2016). Abrahamsson et al. (2014) found that a reduced bacterial diversity during the first month of life is associated with the development of asthma at school age and Zheng et al. (2016) showed in a case-control study that taxa abundances in fecal samples differed between healthy infants and infants with eczema. Apparently, the resulting alterations of the microbiota in early infancy may diminish over time and may not be necessarily detectable in adulthood any longer (Bisgaard et al. 2011).

The distribution of bacterial phyla in our results agrees with some previous findings, but not others. Verrucomicrobia, for example, have not been described as a key component in the equine intestinal microbiome by some reports (Dougal et al. 2013; Venable et al. 2016; Salem et al. 2018), but the larger proportion that we found is in accordance with others (Steelman et al. 2012; Costa et al. 2015; Venable et al. 2016). There might be several reasons for this finding. First of all, different primers may have been used during PCR in the respective studies (Tremblay et al. 2015). Also, the pipeline used during sequence analysis could have an influence. In our study we used the DADA2 pipeline which infers exact amplicon sequence variants (ASVs) instead of the more traditionally employed operational taxonomic units (OTUs) with the advantage that biological differences of even 1 or 2 nucleotides can be resolved (Callahan et al. 2016). Discussion is ongoing about the respective advantages and disadvantages of ASVs and OTUs (Callahan et al. 2017). However, applying the DADA2 algorithm on three mock communities and comparing results to four other common clustering algorithms (UPARSE, MED, mothur and QIIME), Callahan et al. (2016) showed that the overall performance of DADA2 is accurate. Also, it should be kept in mind that the reference reports on the equine intestinal microbiota vary in topicality and that taxa assignment always depends on the existing databases. Regardless of this, the assignment of taxa has no influence on the calculation of alpha and beta diversities, which do not show any significant differences here between affected horses and healthy controls.

As for environmental factors affecting the microbiota we could confirm a significant influence of especially feeding as well as husbandry conditions in terms of stable type and grazing period. Clearly, the three mentioned aspects are not independent of one another. For example, horses that are kept solely on pastures also eat more or even exclusively grass (roughage), because they have unlimited access to it, whereas sport horses are frequently kept in boxes and are also fed higher amounts of concentrate to meet their energy requirements. Figure 4(B–D) illustrate this correlation. A roughly linear change especially in the percentages of Verrucomicrobia (increasing) and Bacteroidetes (decreasing) is apparent in correlation with how much a stable type limits the horse’s access to the outdoors (with “interior box” being at one end of the range to “pasture only” being at the other end). A similar interdependence can be found with regard to the feeding: with increasing amounts of concentrate fed the percentage of Verrucomicrobia decreases and the percentage of Bacteroidetes increases roughly linearly. These results underline the strong impact diet has on the gut microbiota (Albenberg and Wu 2014; David et al. 2014; Harlow et al. 2016; Julliand and Grimm 2017).

Another important observation, which we took into account, is that humans co-habiting the same household frequently share a significant proportion of their microbiota with one another (Song et al. 2013). To investigate this effect in our study population we chose to compare pairwise dissimilarity distances between two samples (Figure 3(A–B)). We found that these pairwise distances are the smallest for horses originated from the same stable, indicating that the microbiota of horses within the same stable is the most similar, i.e., a higher proportion is shared among horses co-inhabiting the same location. This is also in accordance with the findings by Kraemer et al. (2018) that found a similar “location effect” in pigs.

In the face of environmental effects as possible pitfalls, a strength of our study is the close matching of the sampled horse pairs. For every allergic horse we chose a healthy peer from the same stable for comparison and these horse pairs were therefore matched with the main focus on housing and management as well as breed, age and sex, since it is known that the latter factors can also have an effect on the intestinal microbiota (Yatsunenko et al. 2012; Hollister et al. 2014; Gomez et al. 2015).

A possible limitation of our study is the use of rectal swabs, since several other reports have shown that the microbial composition is very site specific (Suchodolski et al. 2005; Zoetendal et al. 2008; Costa and Weese 2012). Furthermore, as Thomas and Fernández-Peñas pointed out (2017), fecal samples best reflect luminal organisms of the large intestine rather than mucosal organisms that may have a greater ability to influence the immune system. We tried to account for this by not only inserting the swab into the rectum, but also gently rubbing it against the rectal mucosa. Rectal swabs were chosen despite this limitation, since they offer the most practicable and noninvasive way of sampling. More invasive methods (e.g., rectal biopsies or cecal fistulation) would not have been an option for this field study in privately owned horses.

## 5. Conclusion

Our observations suggest that hypersensitivity diseases in adult horses are not associated with an alteration of the intestinal microbiota, but the environment of a horse strongly affects the microbial composition. Especially the stable type and feeding exert a strong influence on the bacteria and there is also evidence for a “location effect”, as cohabiting horses share more of their microbiota with each other. These observations contribute to a better understanding of the interrelation between the intestinal microbiota of horses and their environments. The importance of considering the environmental impact on the microbiota is thus highlighted, which is informative for future studies.

## Data Availability

DNA reads have been deposited in NCBI’s Short Read Archive (SRA) repository under accession number PRJNA548852. Additional data files and R codes are available on figshare (DOI: 10.6084/m9.figshare.11973093).

## ABBREVIATIONS

ASV: amplicon sequence variant
bp: base pair
CH: Culicoides hypersensitivity
HOARSI: horse owner assessed respiratory signs index
NMDS: nonmetric multidimensional scaling
OTU: operational taxonomic unit
SEA: severe equine asthma
V4: variable region 4 (of the bacterial 16Sr RNA gene)
